# Influence of Nonnutritive Sucking Habits on the Oral Carriage of *Escherichia coli*

**DOI:** 10.1155/2022/1216727

**Published:** 2022-11-14

**Authors:** Aseel Al Haidar

**Affiliations:** Department of Pedodontics and Preventive Dentistry, College of Dentistry, University of Baghdad, Baghdad, Iraq

## Abstract

**Objectives:**

This study aimed to evaluate the impact of nonnutritive sucking habits on the presence of oral *Escherichia coli*.

**Methods:**

One hundred and twenty children aged 3–5 years old were enrolled in the present case-control study, as follows: 60 children with continuous pacifier and thumb sucking habits (study group) and 60 children without any sucking habits (control group). The children in the two groups were matched in terms of age and gender. Information was gathered from the parents concerning their children using a special sheet. Sterile swabs were taken from both groups and cultured on agar plates. Then, they were subjected to further biochemical tests to identify *E. coli* species. The mean of the *E. coli* count was determined for each child. Descriptive, independent *t*-test and chi-square test statistics were used. The level of significance was set at *p* < 0.05.

**Results:**

The presence of nonnutritive sucking habits was related to a higher carriage of *E. coli* among children.

**Conclusion:**

Nonnutritive sucking habits may act as a predisposing factor that enhances the colonization of oral *E. coli*.

## 1. Introduction

Sucking habits involve frequent neuromuscular activities (conscious and unconscious) and belong to two types: nutritive, in which nutrition can be gained through child sucking; and nonnutritive, in which the child tries to satisfy his/her psychological needs [1–4]. In many countries, nonnutritive sucking habit (NNSH) is a popular norm in infancy and early childhood; however, it is considered abnormal over 3 years of age [2, 5]. While thumb and digit sucking are performed by some children to comfort themselves [1], others soothe and satisfy their unending need to suck by using a dummy (pacifier), which is a plastic, silicon, or rubber teat-like object usually given to infants and toddlers [6]. Although NNSH may provide some beneficial effects, it has more adverse outcomes than positive ones. Some of these negative effects may be related to dentition, such as the development of dental caries and tooth malalignment [7–9]. However, others are related to the child's general health and alteration of the oral and gastrointestinal microbiota, which leads to increased infection [10] and asthma [11]. In addition, NNSH has an adverse effect on the duration of breastfeeding; it may increase the chance of early weaning [7]. Furthermore, NNSH is implicated in the entrance of numerous types of microorganisms into the oral cavity because the objects used for NNSH can act as strong reservoirs of various microorganisms found in the environment [12].

Members of *Enterobacteriaceae*, such as *E. coli*, are mainly found in water, soil, plants, and animals. They are found in the human intestinal canals [13]. However, when found in the oral cavity by NNSH or because of poor hygiene maintenance, they are considered as transient pathogens that might lead to debilitation of general conditions [14].

In the literature, data regarding evaluation of the effect of NNSH and *E. coli* are scarce. It was reported that nail biting and thumb sucking habits played an important role in the oral carriage of *Enterobacteriaceae* [12, 14]. Meanwhile, it was reported that children with pacifier sucking habit had higher carriage of oral *E. coli*, which was associated with diarrhea and increase in gastroenteritis [15, 16]. Other infections caused by *E. coli* may include intestinal infections, cholecystitis, and bacteremia [17].

Not much available data link the presence of *E. coli* in the oral cavity with the NNSH of children (pacifier and thumb sucking habits), which was the premise that the present study was based on. Therefore, this study was conducted to explore whether the presence of oral *E. coli* among children was associated with their NNSH, which was the primary objective of this study, while the secondary objectives were to find if the type of feeding and the rank of the child had any relation to the NNSH and the oral carriage of *E. coli*.

## 2. Materials and Method

### 2.1. Study Design and Sample Population

This case-control study was conducted after getting the ethical approval from the ethical committee of University of Baghdad (Ref. 194320 in September 23, 2020) in accordance with the declaration of the guiding principles of Helsinki [18]. The subjects were recruited from kindergartens in Baqubah city, the center of Diyala Governorate, Iraq, from December 2020 to March 2021. This was a multistage study. The first step was the determination of the case group of the study sample (children with pacifier or thumb sucking habit). The kindergarten's authorities were contacted to explain the purpose of the study. Before the start, the aims of the study were clarified to the parents and a signed permission was obtained from them via a consent form. Healthy children aged 3 to 5 years were the target of the study sample. Children with chronic disease, those who took antibiotics and/or antimycotics for the previous three months, and those whose parents refused to let them participate and/or only partially filled the questionnaire were excluded.

### 2.2. Data Collection

Data collection was done using a questionnaire that was designed to gather the needed information. This questionnaire included questions on demographic information, general health, type of feeding habit, the rank of the child in the family, and occurrence of deleterious NNSH (thumb and pacifier sucking). The questionnaire was sent to the parents, and then the completed questionnaire was collected the next day. Children were classified into two groups according to the presence of oral habits, as follows: (1) the study group, which included children with continuous pacifier and thumb sucking habits; and (2) control group, comprising children from the same kindergarten who had no sucking habits and were age and gender-matched with those in the study group.

### 2.3. Microbiological Examination

Microbiological samples were obtained from children. This was done by swabbing the oral mucosal surface of the children (hard palate, dorsum of the tongue, and cheek and floor of the mouth) using a plane sterile cotton swap (MDIC, Saudi Arabia) [19]. Each swab was streaked on blood agar (Biolife–Haly) and MacConkey agar (Oxoid, England), which is the selective medium for *E. coli*, and incubated aerobically for 24 h at 37°C. Then, the colonies were diagnosed by biochemical tests, which included catalase, coagulase, and oxidase tests. In addition, the API 20E system was used for further identification; this system is commonly used in identifying *E. coli* species from suspected isolates [15, 17, 20]. In this study, method for counting *E. coli* colonies depended on the plate counting method. Serial dilution was done to get clear colony forming unit (CFU) on the MacConkey agar. Then, a direct record to the counts of the colonies with the typical morphology were obtained with regard to the dilution factor and volume cultivated according to the following equation: “(number of colonies multiplied by the dilution factor) divided by the volume of culture plates” [21].

### 2.4. Statistical Analysis

Data were translated into a computerized database structure. Microsoft Excel with Statistical Package for Social Sciences (SPSS) (version 20-computer software, IBM, NY, USA) was used for the data analysis. Data of this study were normally distributed. The dependent variable was the *E. coli* count, whereas the independent one was the NNSH. Variables such as age, gender, type of feeding, and the rank of the child in the family were considered as confounding variables. Descriptive statistics were used. The mean and standard error of the *E. coli* count were determined, and statistical differences were found using *t*-test. However, frequency distribution was used for the categorical variables, and the chi-square test was used to find the statistical difference. The level of significance was set at *p* < 0.05.

## 3. Results

**Figure 1:**
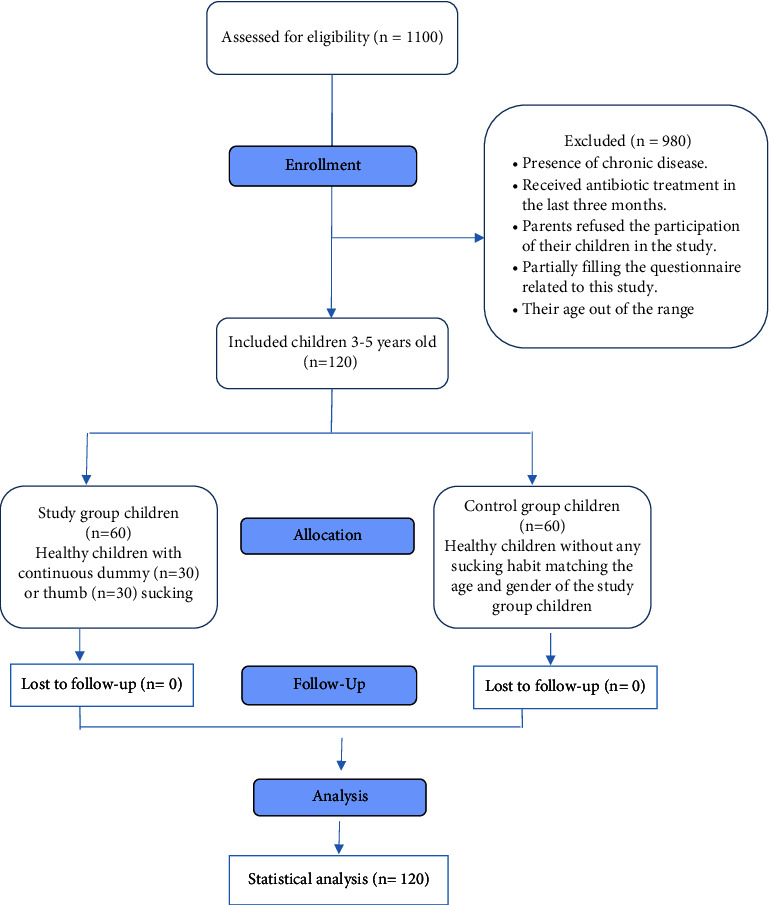
CONSORT flow diagram.

A total of 1100 children were screened, and 120 (56 boys and 64 girls with ages ranging from 3 years old to 5 years old) were enrolled (Figure 1). Among those who practiced NNSH, the carriage rate of *E. coli* was higher with a statistically significant difference. More girl children had NNSH than boys. Meanwhile, girls in the NNSH group showed a higher mean of *E. coli* compared with boys in the same group and with those in the control group (Table 1).

**Figure 2:**
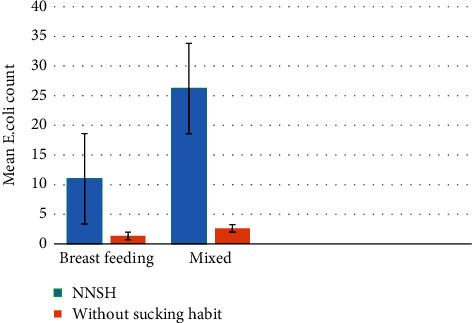
Mean *E. coli* count between the two groups according to type of feeding.

Concerning the method of feeding according to the mean of *E. coli* presence, children in the NNSH group had a higher mean of *E. coli* carriage compared to the children who were without sucking habit with a statistical significant difference. Mixed feeding method (breast and bottle-feeding) was the predominant method of feeding among the children in the NNSH group and those who were without sucking habit (53 and 38, respectively) (Figure 2).

**Figure 3:**
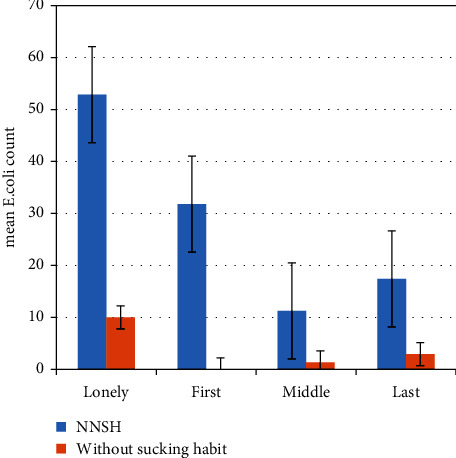
Mean *E. coli* count between the two groups according to the rank of the child.

More children who were the last children of the family had NNSH. However, a higher mean of *E. coli* presence was observed among sole children (Figure 3).

## 4. Discussion

In this study, in all age groups, a higher mean of *E. coli* was present among children in the NNSH group when compared with those without any sucking habit, with a statistical significant difference, especially among those who were 5 years old. This higher presence of microorganisms in the NNSH group was in agreement with the findings of other studies [13, 15]. In the intestinal tract, *E. coli* is usually present as a commensal [22], but it may be present in the oral cavity as a transient microorganism. Its presence in the oral ecosystem is still considered an infection of the oral structures, in spite of its frequent and normal presence in other sites of the human body [23]. Meanwhile, its presence in the oral cavity may increase among children who have NNSH, especially if they neglect oral hygiene or have poor maintenance of personal hygiene, in which case, microbial entry could be via the orofecal route [24]. A potentially alarming effect might result from the entry of enteric microorganisms into the body via the oral cavity [25].

Although the NNSH group had a higher mean of *E. coli*, the carriage rate of *E. coli* by gender revealed that girls had higher mean. This could be due to the fact that girls exhibit emotions and are more sensitive; thus, they may practice NNSH more frequently than boys [26].

Concerning the type of feeding in relation to the mean of *E. coli*, children in the NNSH group had the higher mean of *E. coli* count for both types of feeding found in this study (breast and mixed feeding), and children who were fed by the mixed type of feeding had higher mean of *E. coli* in both groups. This result might be due to the protective impact of breastfeeding against the Gram-negative microbial infection via the enhancement of intraluminal agglutination or by bactericidal effect. Possibly, protection might be related to the inhibition of bacterial attachment to the surface of the oral epithelial tissues [27].

The mean of the *E. coli* count between the two groups according to the rank of the child revealed that a higher mean of *E. coli* was present among children of all ranks within the NNSH group compared with those without sucking habit. However, the results were statistically significant only for the first and the last children. Possibly, those children could practice the habit more frequently than the other children.

## 5. Conclusion

The NNSH showed high correlation with the presence of *E. coli*. They may act as a predisposing factor that enhances the colonization of oral *E. coli* among children. Therefore, stopping these habits may have a significant role in preventing dental as well as oral diseases. Pedodontists should consider the impact of NNSH in increasing the prevalence of *E. coli*, which makes children with this habit more prone to infection. Nevertheless, in an attempt to prevent contamination, children with NNSH should be instructed to maintain proper personal hygiene, and their parents should be instructed to eliminate these habits as soon as possible because of their bad effects on oral and general health.

### 5.1. Limitations of the Study

This study only considered two types of NNSH, namely, thumb sucking and pacifier sucking. Other types, i.e., nail biting, mouth breathing, and bruxism, may also have a deteriorating effect on oral health [23, 28–31]. Other limitations related to the present study were that bacteria other than *E. coli* were not considered. Therefore, further studies are needed for investigating other types of NNSH to assess whether a relationship exists between the oral health status and the spread of *E. coli*.

## Figures and Tables

**Table 1:** Mean *E. coli* count between the nonnutritive sucking habit group and those without any sucking habit according to age and gender.

Variables		*E. coli* count				*t*-Test	df	*p* value^*∗*^
		NNSH		Without sucking habit				
		No.	Mean ± SE	No.	Mean ± SE			
Age (years)	**3**	8	22.38 ± 8.42	8	0.38 ± 0.37	1.83	14	0.001^*∗*^
	**4**	30	13.10 ± 3.56	30	2.33 ± 1.14	2.88	58	0.001^*∗*^
	**5**	22	40.18 ± 12.31	22	2.17 ± 0.78	2.85	42	0.001^*∗*^
	Total	60	24.27 ± 5.16	60	2.17 ± 0.78	4.23	118	0.001^*∗*^

Gender	Boys	28	12.71 ± 3.39	28	1.89 ± 1.03	3.5	54	0.003^*∗*^
	Girls	32	34.37 ± 8.91	32	2.41 ± 1.15	3.6	62	0.001^*∗*^

^*∗*^Significant difference using *t*-test.

## Data Availability

The dataset used in the current study is available from the corresponding author upon request.

## Abbreviations

NNSH: Nonnutritive sucking habit
*E. coli*: *Escherichia coli*.
